# Supplementary material for: Detection of Enriched T Cell Epitope Specificity in Full T Cell Receptor Sequence Repertoires
Source: Front Immunol. 2019 Nov 29;10:2820. doi: 10.3389/fimmu.2019.02820 (PMC6896208; doi:10.3389/fimmu.2019.02820)
Supplement: Supplementary file 1 [file Data_Sheet_1.pdf]

Supplementary Material

Supplemental material S1: Standard TCRex filtering steps

The TCRex webtool performs a few filtering steps on each uploaded TCR dataset: (1) TCR beta sequences with orphon genes, (2) non-canonical TCR beta sequences (i.e. TCRs not starting with cysteine and/or not ending with phenylalanine) and (3) TCR beta sequences containing non-amino acid characters (e.g. a stop codon) or lowercase amino acids are removed. As the immunoSEQ Analyzer format gives information on the frame type of each TCR beta sequence, TCRex retains only the TCR beta sequences from these files that are reported to be in-frame. The IMGT parser ensures that all V/J genes are presented in the IMGT format. This IMGT parser can be switched off when wanted. In this paper, we used the default option where the IMGT parser was activated. Duplicate TCR beta sequences are automatically removed by TCRex. Therefore, all TCR repertoire sizes used in the calculations of the percentage of epitope-specific T cells and the enrichment analyses in this paper refer to the number of unique epitope-specific TCR beta sequences that passed all filtering steps.

Supplemental material S2: Patterns from multiple epitope-specific clusters contribute to the prediction models

TCRex makes use of random forest classifiers to predict epitope-specific TCRs. These machine learning models have the ability to distinguish between different subgroups within the positive (and negative) training data by creating non-linear decision boundaries. As a result, different patterns that are presented by distinct TCR groups, recognizing the same epitope, can contribute to the prediction model. To visualize this, we performed a clustering analysis on the prediction results of the LLWNGPMAV-specific prediction model, which was used extensively in this study. Briefly, we analyzed the LLWNGPMAV-specific training data set with the LLWNGPMAV-specific prediction model using the default BPR threshold (i.e. 0.01%). All 140 training TCRs that passed this filter were clustered using the DBSCAN method (with an epsilon of 4 and a minimal cluster size of 5 TCRs.) To this end, a weighted Euclidian distance measure was calculated with the same TCR features that were used by the prediction model to predict epitope specificity. Hereby, the model-specific feature importances served as weighting factors. In total 133 TCRs were clustered into 4 groups.

The amino acid patterns of these groups are visualized as sequence logos (figure S1). For each position in the TCR sequence, an importance factor was used to scale the sequence logos so that the height of an amino acid character at a specified position represents both the occurrence of this amino acid at this position and the importance of this position in the prediction model. This importance factor was calculated by adding the importances of all features at this position. As can be seen from the final figure S1, the central positions in the TCR sequences are the most important when making epitope-specificity predictions. It is worth to note that these clusters and their corresponding sequence logos are not used by the LLWNGPMAV-specific prediction model, but are a simplified representation of the general patterns that can be detected with this model.

A)

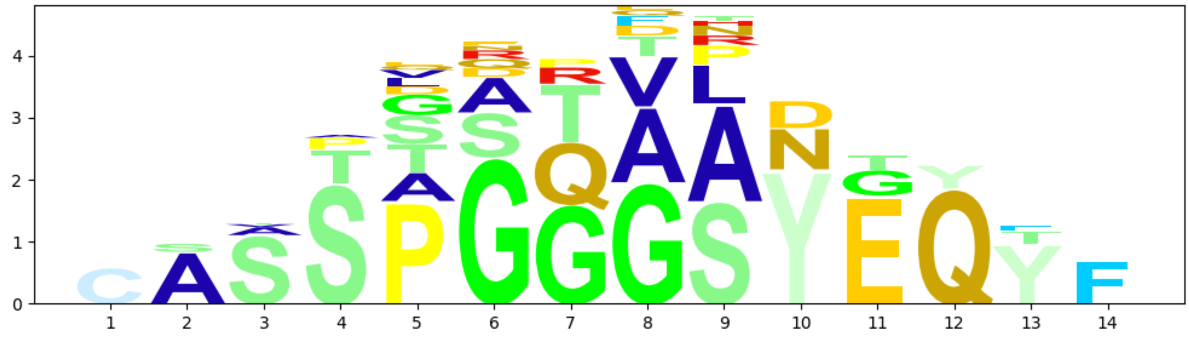

B)

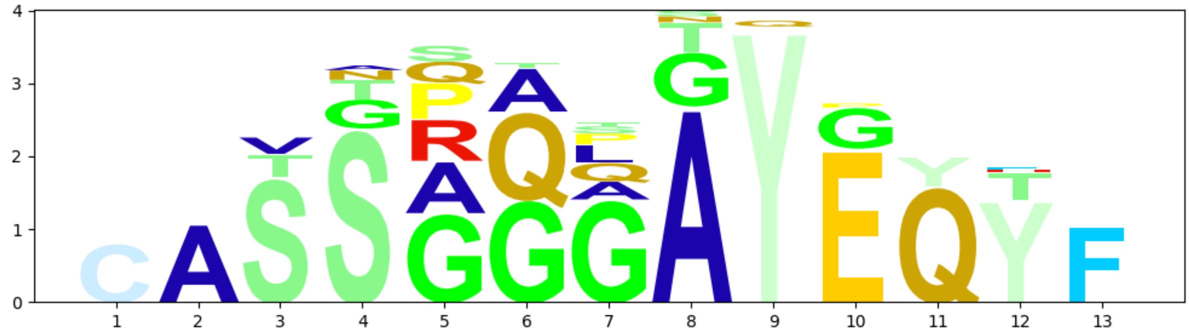

C)

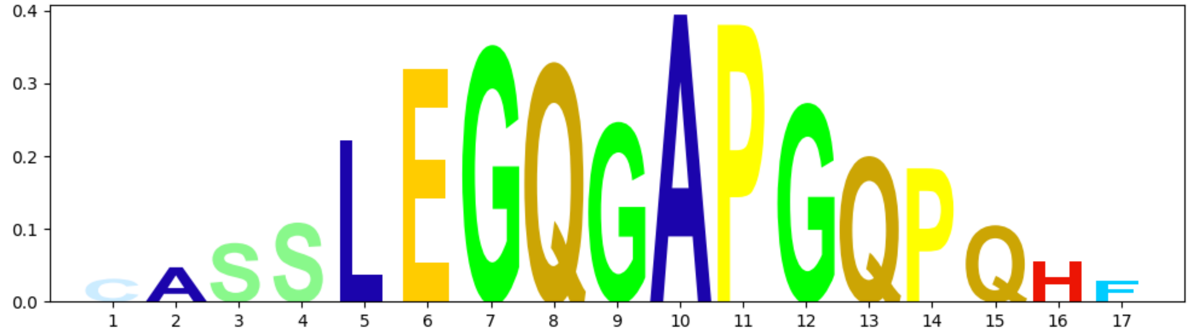

D)

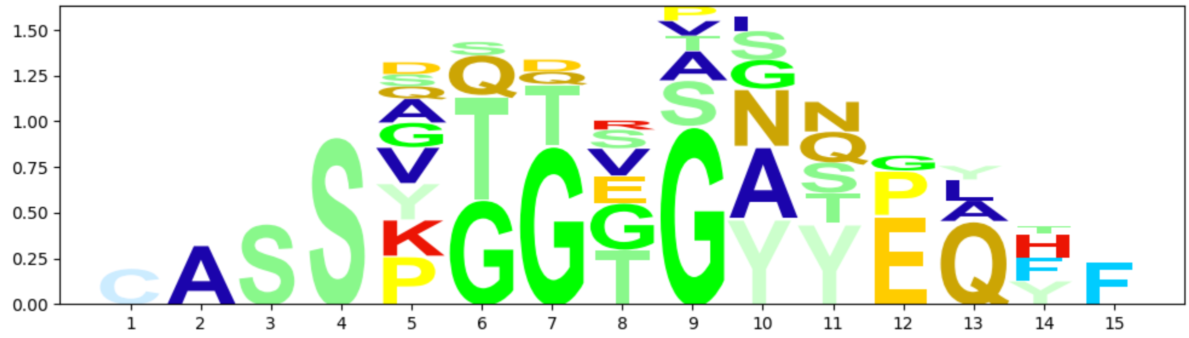

Figure S1: Sequence logos of the clusters present in the LLWNGPMAV-specific training data after filtering with a 0.01% BPR threshold. The clusters contain 59 TCRs (A), 49 TCRs (B), 5 TCRs (C) and 20 TCRs (D).

Supplemental material S3: Web tool instructions

To start a prediction analysis, users are required to upload a file containing TCR data. Different file formats are supported by TCRex, including the immunoSEQ Analyzer[1] and MiXCR (1) output formats. In addition, we propose a simple tab-delimited format that includes the CDR3 amino acid sequence and the V/J genes for all TCR beta sequences following the international ImMunoGeneTics information system (IMGT) notation (2). In case no V/J gene information is available, users are advised to provide the corresponding V/J families.

After uploading the input TCR data file, one or more epitopes can be selected from the database. In this release (version 0.3.0), prediction models for 49 different epitopes are available, including 44 viral and 5 cancer epitopes. With the development and improvement of new TCR sequencing techniques and the rising interest in epitope-specific TCR repertoire analysis, we expect this number to grow rapidly in the near future. The database will be updated regularly with new epitope data made available in the scientific literature. Alternatively, it is also possible to make predictions for epitopes that are not available in the database. To this end, users can upload both their own dataset containing epitope-specific TCR sequences for a single epitope and a TCR test dataset. Both files must follow one of the data formats described above. The epitope-specific TCR sequences are then used to train a new prediction model and the resulting model is subsequently used to make predictions on the uploaded test dataset. To ensure data privacy, these custom models are not made available to other users.

Thereafter, the user can choose whether the V/J genes in the input format need to be changed to the standard IMGT format and select a threshold for the enrichment analysis. This threshold represents the percentage of epitope-specific TCRs in a background dataset. By default, a value of 0.01% is chosen.

When all the required information is submitted, the web tool redirects the user to a web page that gives an overview of all the steps in the prediction process and the current status of the analysis. Once the analysis is finalized, an interactive results summary of the prediction results is given. This allows the user to select a BPR threshold to filter the prediction results. By default, a threshold of 0.01% BPR is used. The filtered results are made available for download as a tab-delimited file. For every TCR sequence provided by the user and for every selected epitope, this file contains a score that represents the probability of epitope-TCR recognition. In addition, each epitope-TCR pair is supplemented with a BPR value, which is used to filter the results. In case the user trained a new prediction model, the results are supplemented with a summary of the performance metrics along with the ROC and PR curves and a visualization of the important features of the learned model. All results are kept available for seven days.

[1] https://www.adaptivebiotech.com/immunoseq

Supplemental material S4: Performance for all trained epitope-specific prediction models

Table S1: Performance metrics of the trained prediction models. For each model, the size of the positive training dataset, the balanced accuracy, the area under the ROC curve (AUC) and the average precision are given. The last column indicates whether the trained model passed all performance criteria.

Source	Epitope	Size positive training dataset	Balanced accuracy	AUC	Average precision	Passed performance criteria
CMV	IPSINVHHY	85	0.7 ± 0.02	0.84 ± 0.04	0.66 ± 0.06	Yes
CMV	MLNIPSINV	73	0.51 ± 0.01	0.61 ± 0.04	0.25 ± 0.04	No
CMV	NLVPMVATV	4812	0.56 ± 0.0	0.72 ± 0.01	0.39 ± 0.01	Yes
CMV	QIKVRVKMV	36	0.5 ± 0.0	0.79 ± 0.05	0.4 ± 0.09	Yes
CMV	QYDPVAALF	41	0.7 ± 0.06	0.82 ± 0.06	0.6 ± 0.06	Yes
CMV	TPRVTGGGAM	258	0.7 ± 0.02	0.88 ± 0.03	0.75 ± 0.04	Yes
CMV	VTEHDTLLY	277	0.54 ± 0.0	0.78 ± 0.02	0.39 ± 0.04	Yes
CMV	YSEHPTFTSQY	74	0.59 ± 0.06	0.93 ± 0.04	0.71 ± 0.06	Yes
DENV1	GTSGSPIVNR	165	0.75 ± 0.05	0.88 ± 0.04	0.76 ± 0.06	Yes
DENV2	GTSGSPIIDK	60	0.61 ± 0.06	0.74 ± 0.08	0.49 ± 0.04	Yes
DENV3/4	GTSGSPIINR	158	0.73 ± 0.02	0.86 ± 0.03	0.75 ± 0.03	Yes
EBV	EPLPQGQLTAY	36	0.64 ± 0.08	0.91 ± 0.06	0.68 ± 0.18	Yes
EBV	GLCTLVAML	1208	0.66 ± 0.01	0.82 ± 0.02	0.59 ± 0.0	Yes
EBV	HPVGEADYFEY	32	0.72 ± 0.08	0.86 ± 0.08	0.68 ± 0.13	Yes
EBV	IVTDFSVIK	46	0.6 ± 0.05	0.83 ± 0.04	0.53 ± 0.05	Yes
EBV	RAKFKQLL	262	0.65 ± 0.02	0.89 ± 0.01	0.69 ± 0.04	Yes
EBV	YVLDHLIVV	103	0.52 ± 0.02	0.76 ± 0.07	0.44 ± 0.09	Yes
HCV	ARMILMTHF	66	0.74 ± 0.07	0.86 ± 0.08	0.69 ± 0.12	Yes
HCV	ATDALMTGY	177	0.75 ± 0.03	0.91 ± 0.04	0.77 ± 0.06	Yes
HCV	CINGVCWTV	131	0.53 ± 0.03	0.75 ± 0.06	0.37 ± 0.11	Yes
HCV	HSKKKCDEL	45	0.77 ± 0.09	0.99 ± 0.01	0.93 ± 0.03	Yes
HCV	KLVALGINAV	65	0.62 ± 0.05	0.73 ± 0.08	0.51 ± 0.14	Yes
HIV	EIYKRWII	185	0.65 ± 0.02	0.75 ± 0.06	0.52 ± 0.04	Yes
HIV	FLKEKGGL	156	0.6 ± 0.03	0.76 ± 0.06	0.48 ± 0.1	Yes
HIV	FPRPWLHGL	120	0.81 ± 0.02	0.93 ± 0.02	0.83 ± 0.03	Yes
HIV	FRDYVDRFYKTLRAEQASQE	367	0.87 ± 0.02	1.0 ± 0.0	0.96 ± 0.01	Yes
HIV	GPGHKARVL	66	0.51 ± 0.02	0.67 ± 0.06	0.31 ± 0.11	No
HIV	HPKVSSEVHI	75	0.69 ± 0.07	0.87 ± 0.08	0.7 ± 0.16	Yes
HIV	IIKDYGKQM	54	0.81 ± 0.07	0.95 ± 0.04	0.84 ± 0.07	Yes
HIV	ISPRTLNAW	58	0.61 ± 0.06	0.84 ± 0.03	0.6 ± 0.12	Yes
HIV	KAFSPEVIPMF	210	0.73 ± 0.02	0.9 ± 0.01	0.71 ± 0.03	Yes
HIV	KRWIILGLNK	396	0.65 ± 0.02	0.85 ± 0.04	0.64 ± 0.08	Yes
HIV	KRWIIMGLNK	75	0.69 ± 0.05	0.85 ± 0.09	0.73 ± 0.12	Yes
HIV	LPPIVAKEI	62	0.79 ± 0.04	0.9 ± 0.05	0.78 ± 0.06	Yes
HIV	QASQEVKNW	31	0.58 ± 0.05	0.6 ± 0.11	0.31 ± 0.12	No
HIV	QVPLRPMTYK	48	0.64 ± 0.04	0.77 ± 0.11	0.51 ± 0.16	Yes
HIV	RFYKTLRAEQASQ	210	0.9 ± 0.03	0.99 ± 0.0	0.96 ± 0.01	Yes
HIV	RLRPGGKKK	31	0.73 ± 0.06	0.89 ± 0.09	0.73 ± 0.2	Yes
HIV	RYPLTFGWCF	30	0.52 ± 0.03	0.72 ± 0.13	0.42 ± 0.12	Yes
HIV	SLYNTVATL	58	0.63 ± 0.03	0.59 ± 0.06	0.4 ± 0.05	No
HIV	TPGPGVRYPL	86	0.68 ± 0.04	0.88 ± 0.03	0.74 ± 0.07	Yes
HIV	TPQDLNTML	159	0.81 ± 0.02	0.94 ± 0.04	0.87 ± 0.06	Yes
HSV2	RPRGEVRFL	63	0.77 ± 0.04	0.92 ± 0.05	0.84 ± 0.08	Yes
HTLV1	SFHSLHLLF	131	0.68 ± 0.03	0.81 ± 0.08	0.63 ± 0.08	Yes
Cancer	NLSALGIFST	111	0.51 ± 0.01	0.66 ± 0.04	0.19 ± 0.03	No

Influenza	GILGFVFTL	3107	0.68 ± 0.01	0.81 ± 0.01	0.59 ± 0.03	Yes
Influenza	LPRRSGAAGA	2137	0.54 ± 0.01	0.84 ± 0.01	0.4 ± 0.02	Yes
Influenza	PKYVKQNTLKLAT	292	0.52 ± 0.01	0.72 ± 0.02	0.36 ± 0.05	Yes
Melanoma	AMFWSVPTV	82	0.59 ± 0.04	0.78 ± 0.04	0.48 ± 0.12	Yes
Melanoma	EAAGIGILTV	266	0.69 ± 0.03	0.9 ± 0.01	0.74 ± 0.01	Yes
Melanoma	ELAGIGILTV	1035	0.54 ± 0.0	0.72 ± 0.01	0.37 ± 0.03	Yes
Melanoma	FLYNLLTRV	61	0.63 ± 0.08	0.87 ± 0.03	0.59 ± 0.1	Yes
Multiple Myeloma	LLLGIGILV	233	0.58 ± 0.01	0.77 ± 0.04	0.44 ± 0.06	Yes
YFV	LLWNGPMAV	474	0.66 ± 0.01	0.79 ± 0.01	0.53 ± 0.03	Yes

Supplemental material S5: MHC background of epitope-specific models

Table S2 gives an overview of the MHC background of all epitopes listed in table S1. For each epitope, it reports the MHC molecules that were associated with the collected TCR beta sequences. Since some of the epitope-specific TCR sequences were collected multiple times due to overlap within or between different sources, we checked whether all these TCRs had the same MHC background. In case multiple MHC molecules were associated with the same TCR, all MHC molecules are reported in the same row and separated with a comma. Epitope-specific TCRs associated with different MHC molecules are highlighted in blue (e.g. one EPLPQGQLTAY-specific TCR is associated with HLA-B*35:01 and HLA-B*35:02). The third column represents the number of unique epitope-specific TCRs that correspond with the MHC background in the second column. The last column gives an overview of the total number of unique TCRs for each epitope, which is identical to the positive training size shown in table S1.

Table S2: Overview of the MHC background of all epitope-specific TCRs in the training dataset

Epitope	MHC background	Number of unique TCR sequences	Total training size per epitope
AMFWSVPTV	HLA-A*02:01	82	82
ARMILMTHF	HLA-B*27	66	66
ATDALMTGY	HLA-A*01	34	177
	HLA-A*01:01	143	
CINGVCWTV	HLA-A*02	58	131
	HLA-A*02, HLA-A*02:01	2	
	HLA-A*02:01	71	
EAAGIGILTV	HLA-A*02	49	266
	HLA-A*02:01	210	
	HLA-A*02:01:48	1	
	unknown	6	
EIYKRWII	HLA-B*08	63	185
	HLA-B*08, HLA-B*08:01	113	
	HLA-B*08:01	9	
ELAGIGILTV	HLA-A*02	968	1035
	HLA-A*02, HLA-A*02:01, HLA-A2	1	
	HLA-A*02, HLA-A2	4	
	HLA-A*02:01	45	
	HLA-A*02:01:48	4	
	HLA-A2	13	
EPLPQGQLTAY	HLA-B*35:01	33	36
	HLA-B*35:01, HLA-B*35:02	1	
	HLA-B*35:01, HLA-B*35:42:01	1	
	HLA-B*35:02	1	
FLKEKGGL	HLA-B*08	73	156

	HLA-B*08, HLA-B*08:01	67	
	HLA-B*08:01	16	
FLYNLLTRV	HLA-A*02:01	61	61
FPRPWLHGL	HLA-B*42:01	120	120
FRDYVDRFYKTLRAEQASQE	HLA-DRA*01&HLA-DRB1*01:01	3	367
	HLA-DRA*01&HLA-DRB1*01:01, HLA-DRA*01&HLA-DRB1*07:01	5	
	HLA-DRA*01&HLA-DRB1*01:01, HLA-DRA*01&HLA-DRB1*07:01, HLA-DRA*01&HLA-DRB1*15:02	1	
	HLA-DRA*01&HLA-DRB1*07:01	12	
	HLA-DRA*01&HLA-DRB1*11:01	4	
	HLA-DRA*01&HLA-DRB1*11:01, HLA-DRA*01&HLA-DRB1*15:02, HLA-DRA*01&HLA-DRB5*01:01, HLA-DRA*01:01&HLA-DRB1*11:01, HLA-DRA*01:01&HLA-DRB1*15:01, HLA-DRA*01:01&HLA-DRB1*15:02, HLA-DRA*01:01&HLA-DRB5*01:01	1	
	HLA-DRA*01&HLA-DRB1*11:01, HLA-DRA*01&HLA-DRB5*01:01	1	
	HLA-DRA*01&HLA-DRB1*11:01, HLA-DRA*01&HLA-DRB5*01:01, HLA-DRA*01:01&HLA-DRB1*11:01, HLA-DRA*01:01&HLA-DRB5*01:01	1	
	HLA-DRA*01&HLA-DRB1*15:02	1	
	HLA-DRA*01&HLA-DRB1*15:02, HLA-DRA*01&HLA-DRB5*01:01	2	
	HLA-DRA*01&HLA-DRB1*15:02, HLA-DRA*01&HLA-DRB5*01:01, HLA-DRA*01:01&HLA-DRB5*01:01	1	
	HLA-DRA*01&HLA-DRB5*01:01	29	
	HLA-DRA*01:01&HLA-DRB1*01:01	29	
	HLA-DRA*01:01&HLA-DRB1*01:01, HLA-DRA*01:01&HLA-DRB1*15:01	3	
	HLA-DRA*01:01&HLA-DRB1*01:01, HLA-DRA*01:01&HLA-DRB5*01:01	2	
	HLA-DRA*01:01&HLA-DRB1*11:01	68	
	HLA-DRA*01:01&HLA-DRB1*11:01, HLA-DRA*01:01&HLA-DRB1*15:01, HLA-DRA*01:01&HLA-DRB5*01:01	1	
	HLA-DRA*01:01&HLA-DRB1*11:01, HLA-DRA*01:01&HLA-DRB5*01:01	9	
	HLA-DRA*01:01&HLA-DRB1*15:01	51	
	HLA-DRA*01:01&HLA-DRB1*15:01, HLA-DRA*01:01&HLA-DRB5*01:01	13	
	HLA-DRA*01:01&HLA-DRB1*15:02, HLA-DRA*01:01&HLA-DRB5*01:01	2	
	HLA-DRA*01:01&HLA-DRB5*01:01	128	
GILGFVFTL	HLA-A*02	1805	3107
	HLA-A*02, HLA-A*02:01	59	
	HLA-A*02, HLA-A*02:01, HLA-A*02:01:48	1	

	HLA-A*02, HLA-A*02:01, HLA-A*02:01:48, HLA-A2	1	
	HLA-A*02, HLA-A*02:01, HLA-A2	41	
	HLA-A*02, HLA-A2	204	
	HLA-A*02:01	568	
	HLA-A*02:01, HLA-A2	268	
	HLA-A2	160	
GLCTLVAML	HLA-A*02	204	1208
	HLA-A*02, HLA-A*02:01	16	
	HLA-A*02, HLA-A*02:01, HLA-A*02:01:48, HLA-A2	1	
	HLA-A*02, HLA-A*02:01, HLA-A*2:01, HLA-A2	1	
	HLA-A*02, HLA-A*02:01, HLA-A2	12	
	HLA-A*02:01	332	
	HLA-A*02:01, HLA-A2	439	
	HLA-A*2:01	6	
	HLA-A2	197	
GPGHKARVL	HLA-B*07:02	66	66
GTSGSPIIDK	HLA-A*11:01	60	60
GTSGSPIINR	HLA-A*11:01	158	158
GTSGSPIVNR	HLA-A*11:01	165	165
HPKVSSEVHI	HLA-B*42:01	75	75
HPVGEADYFEY	HLA-B*35:01	23	32
	HLA-B*35:01, HLA-B*35:08, HLA-B*35:08:01, HLA-B*35:42:01	1	
	HLA-B*35:08	8	
HSKKKCDEL	HLA-B*08:01	44	45
	HLA-B*08:01, HLA-B*08:01:29	1	
IIKDYGKQM	HLA-B*42:01	54	54
IPSINVHHY	HLA-B*35	10	85
	HLA-B*35:01	75	
ISPRTLNAW	HLA-B*57	4	58
	HLA-B*57, HLA-B*57:01, HLA-B*57:03	1	
	HLA-B*57, HLA-B*57:03	5	
	HLA-B*57:01	44	
	HLA-B*57:03	4	
IVTDFSVIK	HLA-A*011	6	46
	HLA-A*011, HLA-A*11	3	
	HLA-A*11	6	
	HLA-A*11:01	31	
KAFSPEVIPMF	HLA-B*57	9	210
	HLA-B*57, HLA-B*57:01	33	
	HLA-B*57, HLA-B*57:01, HLA-B*57:03	4	

	HLA-B*57:01	133	
	HLA-B*57:01, HLA-B*57:03	16	
	HLA-B*57:01, HLA-B*57:06	1	
	HLA-B*57:03	14	
KLVALGINAV	HLA-A*02	65	65
KRWIILGLNK	HLA-B*27	66	396
	HLA-B*27, HLA-B*27:05	54	
	HLA-B*27:05	275	
	HLA-B*27:05:31	1	
KRWIIMGLNK	HLA-B*27	7	75
	HLA-B*27:05	67	
	HLA-B*27:05:31	1	
LLLGIGILV	HLA-A*02	233	233
LLWNGPMAV	HLA-A*02	138	474
	HLA-A*02, HLA-A*02:01	1	
	HLA-A*02:01	335	
LPPIVAKEI	HLA-B*42:01	62	62
LPRRSGAAGA	HLA-B*07:02	1	2137
	HLA-B*07:02, HLA-B7	158	
	HLA-B7	1978	
MLNIPSINV	HLA-A*02	73	73
NLSALGIFST	HLA-A*02	111	111
NLVPMVATV	HLA-A*02	4066	4812
	HLA-A*02, HLA-A*02:01	19	
	HLA-A*02, HLA-A*02:01, HLA-A*02:01:59, HLA-A2	1	
	HLA-A*02, HLA-A*02:01, HLA-A2	2	
	HLA-A*02, HLA-A2	3	
	HLA-A*02:01	581	
	HLA-A*02:01, HLA-A2	7	
	HLA-A*02:01:110	1	
	HLA-A*02:01:98	1	
	HLA-A2	126	
	unknown	5	
PKYVKQNTLKLAT	HLA-DRA*01&HLA-DRB1*01	107	292
	HLA-DRA*01:01&HLA-DRB1*04:01	183	
	HLA-DRA*01:02:03&HLA-DRB1*01:01:01	1	
	HLA-DRA*01:02:03&HLA-DRB1*01:01:01, HLA-DRA*01:02:03&HLA-DRB1*04:01:01	1	
QASQEVKNW	HLA-B*53	8	31
	HLA-B*53, HLA-B*58	1	

	HLA-B*57	5	
	HLA-B*57:01	16	
	HLA-B*58	1	
QIKVRVKMV	HLA-B*08:01	36	36
QVPLRPMTYK	HLA-A*03	28	48
	HLA-A*03, HLA-A*11	5	
	HLA-A*03:01	10	
	HLA-A*11	5	
QYDPVAALF	HLA-A*24:02	41	41
RAKFKQLL	HLA-B*08:01	260	262
	HLA-B*8	2	
RFYKTLRAEQASQ	HLA-DR1	16	210
	HLA-DR1, HLA-DR15	3	
	HLA-DR1, HLA-DR5	2	
	HLA-DR11	47	
	HLA-DR11, HLA-DR15, HLA-DR5	1	
	HLA-DR11, HLA-DR5	3	
	HLA-DR15	100	
	HLA-DR15, HLA-DR5	6	
	HLA-DR5	32	
RLRPGGKKK	HLA-A*03:01	31	31
RPRGEVRFL	HLA-B*07:02	63	63
RYPLTFGWCF	HLA-A*24:02	29	30
	HLA-A*24:02:84	1	
SFHSLHLLF	HLA-A*24:02	131	131
SLYNTVATL	HLA-A*02	1	58
	HLA-A*02, HLA-A*02:01	21	
	HLA-A*02:01	36	
TPGPGVRYPL	HLA-B*07:02	23	86
	HLA-B*42:01	63	
TPQDLNTML	HLA-B*42	5	159
	HLA-B*42:01	114	
	HLA-B*81:01	40	
TPRVTGGGAM	HLA-B*07	18	258
	HLA-B*07, HLA-B*07:02, HLA-B7	1	
	HLA-B*07:02	208	
	HLA-B*07:02, HLA-B7	19	
	HLA-B7	12	
VTEHDTLLY	HLA-A*01:01, HLA-A1	201	277
	HLA-A1	76	
YSEHPTFTSQY	HLA-A*01:01	74	74
YVLDHLIVV	HLA-A*02	10	103

	HLA-A*02:01	93	

Supplemental material S6: Data selection for the leave-on-study-out validation

A leave-one-study-out validation strategy was carried out to assess whether our epitope-specific models are suitable to perform predictions for new studies. This analysis was restricted to viral epitopes for which a large amount of training data (at least 1000 different TCR beta sequences) was collected from different sources. Only three epitopes fulfilled these criteria: NLVPMVATV, GILGFVFTL and GLCTLVAML. For each of these epitopes, we trained epitope-specific models using all collected data except for the TCR beta sequences from one study. The latter was used as an external validation dataset to evaluate the performance of the model on new data. This left out study was selected based on the reported number of epitope-specific TCR cells: (1) studies which reported a limited number of TCR sequences were not eligible as they do not give a good idea of the identification rate of a normal study (2) studies with a large amount of epitope-specific TCRs were also not eligible since we wanted to have a large training dataset. Tables S3-S5 give an overview of the number of epitope-specific TCRs collected from each study following the quality filtering steps as explained in the main text. These numbers are an estimate of the true data size of the studies as duplicate TCRs might still be present, due to an overlap of the different databases and sources we used, and the data were not subjected to the parsing steps that are automatically performed by the TCRex webtool. Therefore, these numbers deviate from the numbers in table 1 of the main text. The selected studies are highlighted in green. All have estimated sizes of approximately 150 TCR sequences.

Table S3: Overview of the references containing GLCTLVAML-specific TCRs

Reference	Number of TCR beta sequences
PMID:28636589	1103
PMID:28636592	132
https://github.com/antigenomics/vdjdb-db/issues/243	130
PMID:19017975	92
PMID:12504586	89
PMID:21555537	44
PMID:10925283	39
PMID:24512815	35
PMID:11046006	33
PMID:19542443	23
PMID:21124993	19
PMID:23267020	17
PMID:25339770	12
PMID:27645996	11
PMID:11592365	7
PMID:16287711	4
PMID:25801351	4

Table S4: Overview of the references containing NLVPMVATV-specific TCRs

Reference	Number of TCR beta sequences
PMID:28423320	4049
PMID:19017975	172
PMID:28636592	152
PMID:28636589	108
PMID:21555537	87
PMID:21374820	63
PMID:16287711	57
PMID:16237109	43
PMID:28623251	33
https://github.com/antigenomics/vdjdb-db/issues/252	29
PMID:24711416	28
PMID:19014475	25
PMID:25801351	20
PMID:25925682	13
PMID:11756174	9
PMID:9971792	9
PMID:19542443	9
PMID:24512815	7
PMID:24069285	7
PMID:23267020	6
PMID:17709536	5
PMID:19403059	5
PMID:25576336	5
PMID:25339770	4
PMID:12616496	3
PMID:21135165	3
PMID:26429912	2
PMID:11930311	2
PMID:19542454	1
PMID:19864595	1

Table S5: Overview of the references containing GILGFVFTL-specific TCRs

Reference	Number of TCR beta sequences
PMID:28423320	2295
PMID:28636589	775
PMID:28636592	406
PMID:28300170	153
PMID:25609818	101
PMID:29483513	32
PMID:27645996	29
PMID:25339770	26
PMID:28250417	22
PMID:25801351	16
PMID:7807026	12
PMID:18275829	1
PMID:12796775	1

Supplemental material S7: Calculation of the performance metrics for the leave-on-study-out validation

Following standard formulas were used to calculate the performance metrics in table 1 of the main text.

$$Accuracy = \frac{TP + TN}{TP + TN + FP + FN}$$

$$Sensitivity = \frac{TP}{TP + FN}$$

$$Specificity = \frac{TN}{TN + FP}$$

$$Precision = \frac{TP}{TP + FP}$$

With:

- FP = number of identified TCRs in the cancer dataset
- TN = number of TCRs in the cancer dataset that were classified as non-epitope binding
- TP = number of identified TCRs in the epitope-specific external validation dataset
- FN = number of TCRs in the epitope-specific external validation dataset that were classified as non-epitope binding

Supplemental material S8: Public versus TCRex identified LLWNGPMAV-specific TCRs

Table S6: Number of LLWNGPMAV-specific TCR sequences in the post-vaccination PBMC samples of (3)**.** For each volunteer from (3) are given: the number of LLWNGPMAV-specific TCRs in the post-vaccination repertoire identified with TCRex (0.01% BPR), the number of training LLWNGPMAV-specific TCRs in the post-vaccination repertoire and the corresponding overlap (i.e. the number of training TCR sequences that were identified with TCRex).

Volunteer	TCRs identified by TCRex	Training TCRs	Overlap
1	32	13	4
2	65	19	7
3	87	34	9
4	34	11	2
5	63	20	7
6	32	12	4
7	11	7	2
8	27	12	4
9	18	6	1

Table S7: Number of LLWNGPMAV-specific TCR sequences in the post-vaccination PBMC samples of (4)**.** For each volunteer from (4) are given: the number of LLWNGPMAV-specific TCRs in the post-vaccination repertoire identified with TCRex (0.01% BPR), the number of training LLWNGPMAV-specific TCRs in the post-vaccination repertoire and the corresponding overlap (i.e. the number of training TCR sequences that were identified with TCRex).

Volunteer	TCRs identified by TCRex	Training TCRs	Overlap
P1	92	31	12
P2	110	36	9
Q1	79	26	12
Q2	92	33	12
S1	170	48	21
S2	273	53	26

References

1. Bolotin DA, Poslavsky S, Mitrophanov I, Shugay M, Mamedov IZ, Putintseva E V., Chudakov DM. MiXCR: Software for comprehensive adaptive immunity profiling. *Nat Methods* (2015) **12**:380–381. doi:10.1038/nmeth.3364
2. Lefranc MP, Giudicelli V, Duroux P, Jabado-Michaloud J, Folch G, Aouinti S, Carillon E, Duvergey H, Houles A, Paysan-Lafosse T, et al. IMGT, the international ImMunoGeneTics information system 25 years on. *Nucleic Acids Res* (2015) **43**:D413–D422. doi:10.1093/nar/gku1056
3. Dewitt WS, Emerson RO, Lindau P, Vignali M, Snyder TM, Desmarais C, Sanders C, Utsugi H, Warren EH, McElrath J, et al. Dynamics of the Cytotoxic T Cell Response to a Model of Acute Viral Infection. *J Virol* (2015) **89**:4517–4526. doi:10.1128/JVI.03474-14
4. Pogorelyy M V., Minervina AA, Touzel MP, Sycheva AL, Komech EA, Kovalenko EI, Karganova GG, Egorov ES, Komkov AY, Chudakov DM, et al. Precise tracking of vaccine-responding T cell clones reveals convergent and personalized response in identical twins. *PNAS* (2018) **115**:12704–12709. doi:10.1073/pnas.1809642115
